# Characterization of the Copper Transporters from *Lotus* spp. and Their Involvement under Flooding Conditions

**DOI:** 10.3390/ijms20133136

**Published:** 2019-06-27

**Authors:** Francisco J. Escaray, Cristian J. Antonelli, Guillermo J. Copello, Sergi Puig, Lola Peñarrubia, Oscar A. Ruiz, Ana Perea-García

**Affiliations:** 1 Instituto Tecnológico de Chascomús (INTECh), UNSAM/CONICET, Avda. Intendente Marino Km. 8.2, Chascomús, Buenos Aires 7130, Argentina; franescaray@gmail.com (F.J.E.); antonelli@intech.gov.ar (C.J.A.); ruiz@intech.gov.ar (O.A.R.); 2 Departament de Bioquímica i Biologia Molecular, Estructura de Recerca Interdisciplinar en Biotecnologiaia i Biomedicina (ERI BIOTECMED), Universitat de València. Burjassot, 46100 Valencia, Spain; Lola.Penarrubia@uv.es; 3 Instituto de Fisiología Vegetal (INFIVE), Universidad Nacional de La Plata (UNLP), Consejo Nacional de Investigaciones Científicas y Técnicas (CONICET), La Plata, Buenos Aires 1900, Argentina; 4 Instituto de Química y Metabolismo del Fármaco (IQUIMEFA), Universidad de Buenos Aires (UBA), Consejo Nacional de Investigaciones Científicas y Técnicas (CONICET), Ciudad Autónoma de Buenos Aires, Buenos Aires C113AAD, Argentina; gcopello@ffyb.uba.ar; 5 Departamento de Química Analítica y Fisicoquímica, Facultad de Farmacia y Bioquímica, Universidad de Buenos Aires (UBA), Ciudad Autónoma de Buenos Aires, Buenos Aires C113AAD, Argentina; 6 Instituto de Agroquímica y Tecnología de los Alimentos, Centro Superior de Investigaciones Científicas, IATA-CSIC, Paterna, 46980 Valencia, Spain; spuig@iata.csic.es

**Keywords:** copper transporters, legumes, flooding, forage

## Abstract

Forage legumes are an important livestock nutritional resource, which includes essential metals, such as copper. Particularly, the high prevalence of hypocuprosis causes important economic losses to Argentinian cattle agrosystems. Copper deficiency in cattle is partially due to its low content in forage produced by natural grassland, and is exacerbated by flooding conditions. Previous results indicated that incorporation of *Lotus* spp. into natural grassland increases forage nutritional quality, including higher copper levels. However, the biological processes and molecular mechanisms involved in copper uptake by *Lotus* spp. remain poorly understood. Here, we identify four genes that encode putative members of the *Lotus* copper transporter family, denoted COPT in higher plants. A heterologous functional complementation assay of the *Saccharomyces cerevisiae ctr1∆ctr3∆* strain, which lacks the corresponding yeast copper transporters, with the putative *Lotus* COPT proteins shows a partial rescue of the yeast phenotypes in restrictive media. Under partial submergence conditions, the copper content of *L. japonicus* plants decreases and the expression of two *Lotus COPT* genes is induced. These results strongly suggest that the *Lotus* COPT proteins identified in this work function in copper uptake. In addition, the fact that environmental conditions affect the expression of certain *COPT* genes supports their involvement in adaptive mechanisms and envisages putative biotechnological strategies to improve cattle copper nutrition.

## 1. Introduction

Copper (Cu) is an essential micronutrient for all living organisms because it participates as a cofactor in biological processes, such as respiration, photosynthesis, carbon and nitrogen metabolism, and oxidative stress protection [1]. Cu plays a dual role since it is an important redox transition metal, but it is toxic in excess [2]. Hypocuprosis or Cu deficiency is one of the main nutritional disorders in grazing livestock around the world. Cattle affected by Cu deficiency were detected in several countries of South and Central America [3]. Specifically, the incidence of hypocuprosis has been associated with flooding periods at the Flooding Pampa (FP) in Argentina [4]. Particularly, calves and pregnant cattle are the most affected livestock categories because they have the highest food requirements of the cattle farming. Two types of Cu deficiency have been described: (a) Primary or simple, which is caused by a low Cu presence in the diet; and (b) secondary or conditioned, when the presence of Cu is adequate, but the presence of other metals interferes with Cu digestive absorption [5]. 

According to a global study carried out by the Food and Agriculture Organization of the United Nations (FAO), the nutrient status of Cu in Argentina soils, and consequently in plants including forage, is generally low [4]. FP is an extensive region characterized by alkaline-saline soils and waterlogging periods due to the absence of drainage slopes [6]. During rainy years, flood periods are especially common from autumn to spring [6,7]. As a consequence, species richness decreases in flood-prone regions with alkaline or saline soils [7]. Under these conditions, the main food source for cattle are natural grasslands [6], which produce a low quality forage since they are dominated by grasses, with very few legumes. In this environment, *Lotus tenuis* (Lt), a naturalized species, has a key role improving the sustainability of cattle production [8]. Moreover, it has been described that Lt better supports flooding treatment than other evaluated species of the *Lotus* genus [9,10,11]. 

Flooding stress affects the development of morphological traits, such as adventitious roots, in order to improve anchoring and assimilation of water and nutrients [12,13]. In addition, the oxygen concentration decreases in this reductant environment, affecting the bioavailability of Cu, among other micronutrients, since it restricts uptake [14,15]. Moreover, reductant environment conditions enhance the solubility of other metals, such as iron (Fe) or molybdenum (Mo), which increases its uptake and competition with Cu entrance, exacerbating Cu deficiency [16]. In ruminants, Mo and Fe decrease Cu digestive absorption [17]. Mo is an essential micronutrient for plants, since is a key cofactor of the nitrate reductase and nitrogenase enzymes. However, high levels of Mo in the cattle diet induce toxicity per molybdenosis and induced hypocuprosis [18]. In this sense, Mo binds to sulfide forming thiomolybdates, which are able to complex the Cu, and decreasing its digestive absorption [19]. In the case of Fe, the decrease of Cu levels observed in cattle tissues (liver or blood) has been suggested to be caused by Fe-S complexes’ formation that antagonizes Cu absorption in the rumen or at the intestinal level, through competition of divalent metals for the Divalent metal transporter 1 (DMT1) [20,21]. 

Under Cu-deficient conditions, the conserved family of CTR/COPT high affinity transporters mediate Cu (I) acquisition into the cytosol [22,23,24]. The CTR/COPT proteins contain three transmembrane domains (TMDs), with the amino-terminal region facing the extracellular space and the carboxy-terminal end towards the cytosol. Some CTR/COPT proteins contain methionine-rich domains at the amino-terminal end that function in Cu binding. After binding to these motifs, the metal is transported through the pore generated by the methionine-rich TMD2 homotrimer and delivered to the cysteine residues at the cytosolic carboxy-terminus. Once there, Cu is driven to its target proteins through specific metallochaperones that complex the metal to avoid its toxicity and undesirable interactions. In *A. thaliana*, six members of the COPT family, denoted COPT1–6, have been characterized and classified according to different experimental approaches [22]. Under Cu deficiency *COPT1*, *COPT2*, and *COPT6* genes are induced at the transcriptional level, while *COPT3* and *COPT5* are not [22]. In agreement with the above, *COPT1*, *COPT2*, and *COPT6* possess Cu response elements (CuRE, GTAC) [25,26]. The transcriptional factor, Squamosa promoter binding protein like 7 (SPL7), is essential for regulation under Cu deficiency by binding to these GTAC motifs [27,28]. The subcellular localization in *Arabidopsis* protoplasts allows the division of the COPT family into plasma membrane transporters (COPT1, COPT2, and COPT6) and transporters of intracellular compartments (COPT3 and COPT5) [25,26,29,30]. Functional complementation of the corresponding *S. cerevisiae* strains, divides the COPT family into transporters that function in the extracellular Cu import through the plasma membrane (COPT1, COPT2, and COPT6 [22,25,26,29,31] and transporters that mediate intracellular transport (COPT3 and COPT5), which only partially revert the phenotype of yeast mutant strains [22,30,31,32]. Tissue localization by GUS reporter expression driven by its promoters allowed the definition of the specificity of their expression pattern and the characterization of the corresponding T-DNA insertion mutants to give clues about their functions. Briefly, COPT1 plays an important role in root Cu uptake and in pollen development [31,32]. COPT2 is the most highly expressed Cu transporter in different tissues and its function interacts with Fe and phosphate homeostasis [25]. COPT6 is expressed mostly in the aerial part, especially in vascular bundles and reproductive tissues [26,33]. COPT3 participates in pollen grains and vascular bundles’ Cu transport [33]; and COPT5 is mainly located in the tonoplast of vascular bundles of the root cells and in the siliques, and functions in Cu storage in the vacuoles [30], also affecting Fe homeostasis in this compartment [34]. Some of these proteins have been characterized in the species of agronomical interest. Thus, seven genes encoding Cu transporters have been described in rice (*Oryza Sativa*) [35]; eight members in grapevine (*Vitis vinifera*) [36]; three proteins in maize [37]; and seven in *Populus trichocarpa* [37]. Recently, the Copper Transporter 1 (MtCOPT1) protein from the legume *Medicago truncatula*, has been functionally characterized as a Cu transporter from the apoplast into nodule cells [13]. However, high affinity Cu transporters in *Lotus* spp. still remain uncharacterized and, in general, little is known about these transporters in legumes. Here, the *L. japonicus* COPT protein family has been characterized and their expression response to partial submergence conditions has been evaluated in order to dissect their putative roles in Cu uptake. 

## 2. Results

### 2.1. Lotus Species Differentially Respond to Partial Submergence Conditions

**Figure 1:**
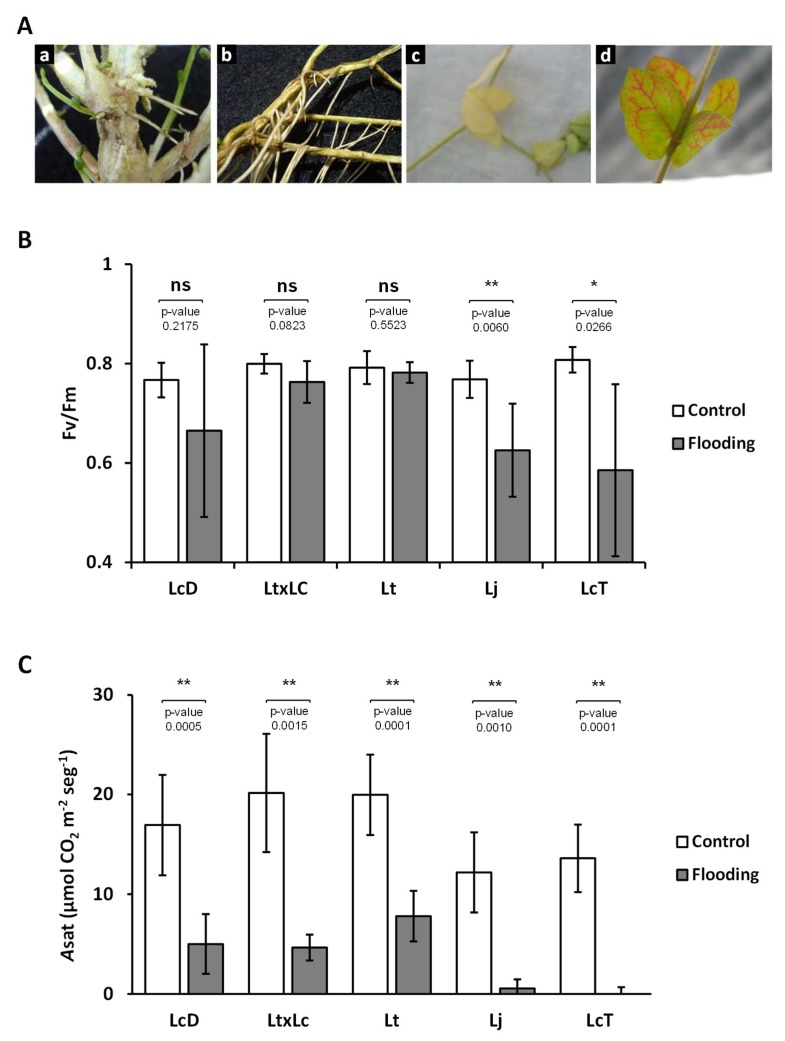
Effects of partial submergence in different species of the *Lotus* genus. (**A**) Representative photographs of *L. corniculatus* diploid (LcD) plants after 42 days of treatments (control and flooding). Plants were grown under partial submergence described in Material and Methods and details of different symptoms are shown: (a) Thickening; (b) adventitious roots; (c) chlorosis; and (d) reddish veins. (**B**) The maximum quantum yield of primary Photosystem II (PSII) photochemistry (*F*v/*F*m). (**C**) Net photosynthetic rate under saturating irradiance (*Asat*). Values are means ± SD of six biological replicates. Between treatments for each genotypes, one asterisk (*P* < 0.05) and two asterisks (*P* < 0.01) over the bars mark a significant difference; ns indicate no significant difference (*P* > 0.05). LcD: *L. corniculatus* diploid, Lt: *Lotus tenuis*, LtxLc: *L. tenuis* × *L. corniculatus*, Lj: *L. japonicus* and LcT: *L. corniculatus* tetraploid plants.

In order to study the effects of flooding stress into different accessions of the *Lotus* genus, one-month old plants were cultivated under partial submergence for 42 days under greenhouse experimental conditions mimicking soil and irrigation flooding suffered in the FP. After a partial submergence treatment, different symptoms were observed in the evaluated *Lotus* spp. Among them, as shown in Figure 1A, was the thickening of the main stem base (a), the presence of adventitious roots (b), the chlorosis of basal leaves (c), and a reddish coloration of the veins (d). Even if all evaluated genotypes showed similar symptoms when grown under this treatment, plants from *Lotus japonicus* (Lj) and *L. corniculatus* tetraploid (LcT) showed the most marked effects compared to others under similar conditions. 

Chlorophyll fluorescence was measured at the end of the treatment to estimate PSII activity in *Lotus* spp. plants under partial submergence and control conditions. The maximum quantum yield of PSII (*F*m/*F*v) of control plants was around 0.80 (Figure 1B). When subjected to submergence treatment, plants from Lj and LcT accessions showed lower *F*m/*F*v values than controls. *Lotus corniculatus* diploid (LcD) treated plants showed lower *F*m/*F*v values than the control, although they were not statistically significant (Figure 1B). Finally, no differences in *L. tenuis* (Lt) and *L. tenuis* x *L. corniculatus* (LtxLc) plants were detected under submergence treatment (Figure 1B).

The gas exchange measurements showed that the partial submergence treatment considerably affected the net photosynthesis rate (*Asat*) in all *Lotus* spp. accessions evaluated (Figure 1C). However, the detrimental effect of the treatment was different among accessions. Whereas Lj and LcT were the most affected plants, showing very low *Asat* values, Lt, LcD, and LtxLc were less affected (61%, 70%, and 77% of reduction, respectively) (Figure 1C). Stomatal conductance (*gs*) and the performance index (PIabs) were also affected by treatment in almost all *Lotus* spp. accessions, with LcD the only plants maintaining values similar to the control condition (Table S3). In the same way as *Asat*, the *gs* in Lj and LcT was more affected than in Lt and LtxLc plants (Table S3). Finally, other anatomical features, such as biomass production, were measured under the flooding treatment and all the accessions showed a decrease in accumulated biomass compared to controls (Figure S1 and Table S3).

### 2.2. Partial Submergence Conditions Affected Plant Metal Content in Lotus Species

**Figure 2:**
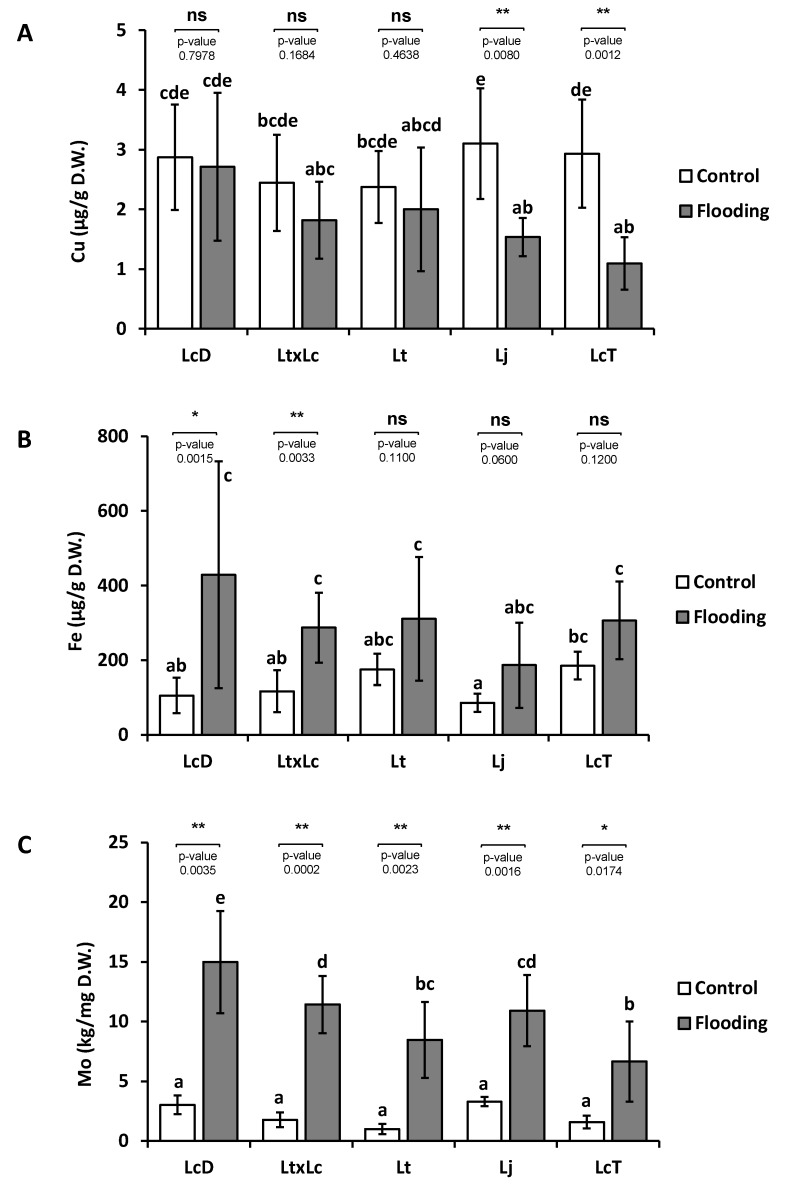
Endogenous Cu, Fe, and Mo concentrations in different species from the *Lotus* genus. Cu (**A**), Fe (**B**), and Mo (**C**) concentrations were measured from the shoots of LcD: *L. corniculatus* diploid. Lt: *Lotus tenuis*; LtxLc: *L. tenuis* × *L. corniculatus*. Lj: *L. japonicus* and LcT: *L. corniculatus* tetraploid plants. Plants were grown under partial submergence described in Material and Methods. Values are means ± SD of six biological replicates. DW: Dry weight. Different letters above the bars represent significant differences among all the means (*P* < 0.05). Between treatments for each genotypes, one asterisk (*P* < 0.05) and two asterisks (*P* < 0.01) over the bars mark a significant difference; ns indicate no significant difference (*P* > 0.05).

In order to evaluate the effect of the flooding treatment on endogenous metal levels, Cu, Fe, and Mo contents were determined in *Lotus* spp. plants under control and partial submergence conditions (Figure 2). Under control conditions, Cu and Mo contents were similar between genotypes (Figure 2A,C). In the case of Fe content, LcT plants showed slightly higher content than Lj under control conditions. However, no differences among other genotypes were observed (Figure 2B).

The flooding treatment affected the Cu concentration in Lj and LcT ecotypes, showing significant lower levels of this metal under stress compared to the control condition (Figure 2A). Cu levels were not affected by this treatment in the other genotypes: LcD, LtxLc, and Lt plants. Interestingly, a complementary pattern was observed for Fe concentration; LcD and LtxLc treated plants showed statistically significant higher levels than under control conditions. Meanwhile, no differences in Lt, Lj, and LcT plants were detected when the treatment was compared to control conditions (Figure 2B). Finally, Mo content was largely affected by partial submergence (Figure 2C). Thus, levels of this metal were higher in all the samples from treated plants compared to control conditions.

Comparing genotypes under partial submergence, samples from LcD plants showed higher Cu levels than those from Lj and LcT (Figure 2A). No differences in Fe concentration were observed between the evaluated *Lotus* genotypes under the submergence treatment. Finally, as well as the observed Cu content, the LcD plants under partial submergence showed higher Mo concentrations than the other evaluated genotypes under similar conditions. Moreover, lower Mo values were determined in samples from LcT and Lt treated plants (Figure 2C).

### 2.3. The Predicted Family of Copper Transporters from Lotus japonicus

Based on gene functional annotation and considering the (InterPro) IPR007274 Ctr copper transporter code, four putative CTR/COPT sequences were identified in the *Lotus japonicus* genome (Miyakogusa.jp2.5 database, http://www.kazusa.or.jp/lotus/release2/). Accession numbers correspond to Gene Bank accession number MN065182 (*LjCOPT1*), *chr1.LjT44L17.20.r2.d* (*LjCOPT2*), *chr1.CM0133.1180.r2.m* (*LjCOPT3*), and *chr5.CM0040.160.r2.d* (*LjCOPT4*).

**Figure 3:**
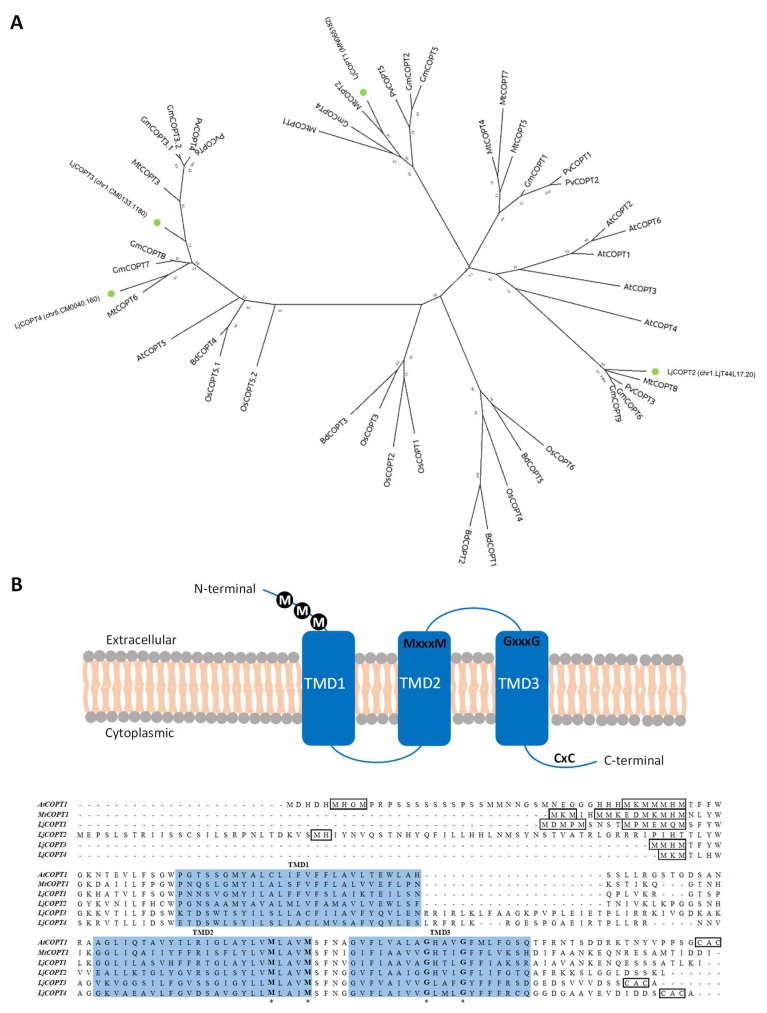
Copper transporter protein family in *Lotus* species. (**A**) Evolutionary analysis by the maximum likelihood method of *Lotus* spp. Copper transporters (COPT) and representative plant COPT homologs (bootstrap consensus unrooted tree inferred from 1000 replicates). (**B**) Proposed topology of *Lotus* spp. COPTs and their amino acid sequences. Methionine and/or hystidine rich motifs (M) at the N-terminal region and CxC residues at the C-terminal are indicated in black blocks. Putative transmembrane domains (TMDs) are indicated in blue squares and MxxxM and GxxxG motifs are indicated by asterisks.

The deduced amino acid sequences obtained for the COPTs genes from *L. japonicus* (LjCOPTs) were aligned to COPTs sequences from different dicotyledons (*Medicago truncatula*, *Glycine Max*, *Phaseolus vulgaris*, and *Arabidopsis thaliana*) and monocotyledons (*Oryza sativa* and *Brachypodium distachyon*) plants species and used to construct a phylogenetic tree (Figure 3A). LjCOPTs clustered into three different branches, and LjCOPT1 was located in a cluster shared by COPT legume sequences: Two from *Medicago truncatula* (MtCOPT1 and MtCOPT2), three from *Glycine max* (GmCOPT2, GmCOPT4, and GmCOPT5), and one from *Phaseolus vulgaris* (PvCOPT5). LjCOPT2 was located in another legume exclusive cluster, including MtCOPT8, PvCOPT3, GmCOPT6, and GmCOPT9 sequences. Finally, LjCOPT3 and LjCOPT4 were positioned close to legume sequences, such as MtCOPT3, MtCOPT6, GmCOPT7, and GmCOPT8, in another well-supported cluster apart from LjCOPT1 and LjCOPT2 (Figure 3A) and next to AtCOPT5 from *Arabidopsis thaliana*. 

Within the four predicted amino acid LjCOPT sequences, the highest sequence identity was observed between LjCOPT3 and LjCOPT4 (59.1%). However, the similarity was less than 30% among other LjCOPTs’ sequences (Table S4). 

The amino acid sequence alignment of *L. japonicus* COPT proteins showed the conserved motifs previously described in other CTR/COPT members. Thus, all the LjCOPT members possess three predicted transmembrane domains (TMDs) and the conserved MxxxM and GxxxG motifs located within TM2 and TM3, respectively [23] (Figure 3B). Also, all, except LjCOPT2, exhibited the conserved methionine and/or histidine rich motifs (Met/His motifs) at their amino-terminal region, which have been suggested to sequester and stabilize Cu (I) [23] (Figure 3B). Finally, LjCOPT3 and LjCOPT4 both contain the C-terminal cysteine-rich CXC motif, which was suggested as being essential either to bind Cu into the cytosolic part from metallochaperones, or to downregulate the transport to avoid Cu toxicity [38,39].

### 2.4. Functional Complementation of Lotus japonicus COPTs

**Figure 4:**
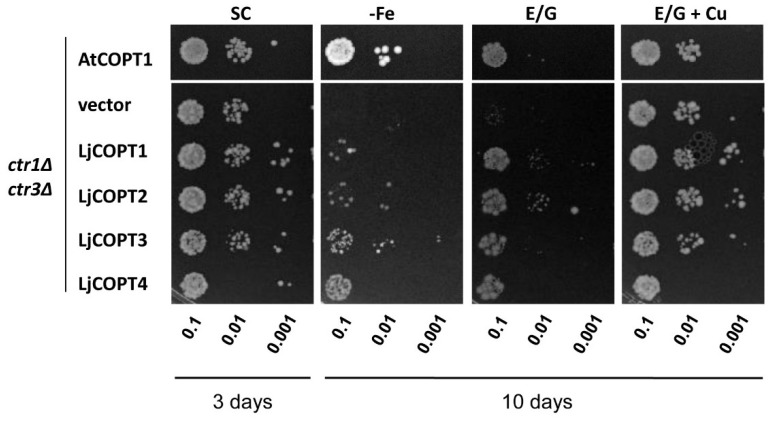
Functional complementation of *copper transporter 1 and copper transporter 3* (*ctr1∆ctr3∆*) *Saccharomyces cerevisiae* mutant. The *ctr1∆ctr3∆* mutant was transformed with empty vector (p426GPD; negative control). The same vector expressing *Arabidopsis thaliana* AtCOPT1 (positive control) and LjCOPT1, LjCOPT2, LjCOPT3, or LjCOPT4. Cells were grown at 30 °C on glucose (SC-ura) plates or on glucose plus Ferrozine (300 µM), ethanol/glycerol (E/G) or ethanol/glycerol plus CuSO_4_ (100 µM) for 3 to 10 days.

In order to evaluate the function of the LjCOPT1–4 proteins in Cu transport, a complementation analysis was performed in the MPY17 (*ctr1∆ctr3∆*) *S. cerevisiae* mutant (Figure 4). The four putative *Lotus* Cu transporters *LjCOPT1*, *LjCOPT2*, *LjCOPT3*, and *LjCOPT4* genes were amplified by specific oligonucleotides (Table S1) and cloned into the multicopy expression vector p426GPD (*glyceraldehide*-3-phosphate dehydrogenase gene promoter). Transformants were able to grow on Synthetic Complete media (SC) (Figure 4). Under Fe deficiency, Fe uptake is mediated by a Cu-dependent high affinity Fe complex [40]. Thus, when the medium was supplemented with the Fe^2+^-chelator Ferrozine, the *ctr1∆ctr3∆* lack of growth when containing the empty vector was restored when transformed with AtCOPT1 (Figure 4). In the case of LjCOPT1, LjCOPT2, LjCOPT3, and LjCOPT4, *ctr1∆ctr3∆* cell growth was partially restored (Figure 4).

To further evaluate the ability of the COPT family members to grow under non-fermentable carbon sources (ethanol/glycerol), *ctr1∆ctr3∆* growth was analyzed under these conditions (Figure 4). Yeast growth in non-fermentable carbon sources depends on the correct Cu distribution to the mitochondrial cytochrome oxidase [24]. Thus, we observed a restored growth of the *ctr1∆ctr3∆ S. cerevisiae* mutant when it was complemented with LjCOPT1, LjCOPT2, LjCOPT3, and LjCOPT4 similar to the growth observed when transformed with AtCOPT1 (Figure 4). As expected, the growth of *ctr1∆ctr3∆* cells was restored when the medium was supplemented with excess Cu since Cu uptake takes place through transporters other than COPTs under these conditions.

### 2.5. COPTs Gene Expression in Lotus japonicus 

**Figure 5:**
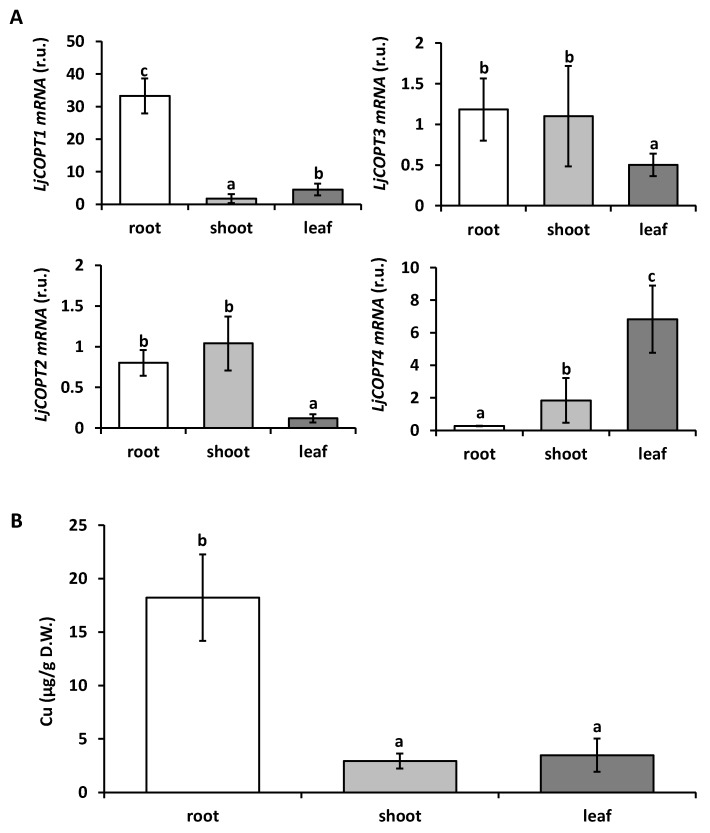
Gene expression and copper content in different tissues of *Lotus japonicus*. (**A**) Gene expression of *LjCOPT1*, *LjCOPT2*, *LjCOPT3*, and *LjCOPT4* was analyzed by RT-qPCR and normalized to the transcript levels of the *Elongation Factor* (*EF-1α*) gene. The stem sample was used as a reference. (**B**) Cu content was measured in root, stem, and leaf samples from *L. japonicus*. Plants were grown under control conditions as described in Material and Methods. DW: Dry weight. Values are means ± SD of three and six biological replicates, respectively. Different letters above the bars represent significant differences among all the means (*P* < 0.05).

To assess the involvement of *COPTs* in Cu uptake in *L. japonicus*, the *LjCOPTs* gene expression and the endogenous Cu content were measured in distinct tissues as shown in Figure 5. The *LjCOPTs* gene expression patterns were detected in different tissues from *L. japonicus* plants (Figure 5A). *LjCOPT1* was more expressed in roots than in stems and leaves, whereas *LjCOPT2* and *LjCOPT3* were similarly expressed in roots and stems, but lower in leaves (Figure 5A). Finally, in the case of *LjCOPT4*, the highest expression was detected in the leaves and very low levels were observed in roots (Figure 5A).

Analyses performed by Atomic Absorption (AA) spectroscopy showed that Cu levels were about 10 times higher in roots than in stems and leaves from *L. japonicus* plants grown under optimal conditions (Figure 5B).

### 2.6. Regulation of Metal Homeostasis Genes under Partial Submergence Conditions

**Figure 6:**
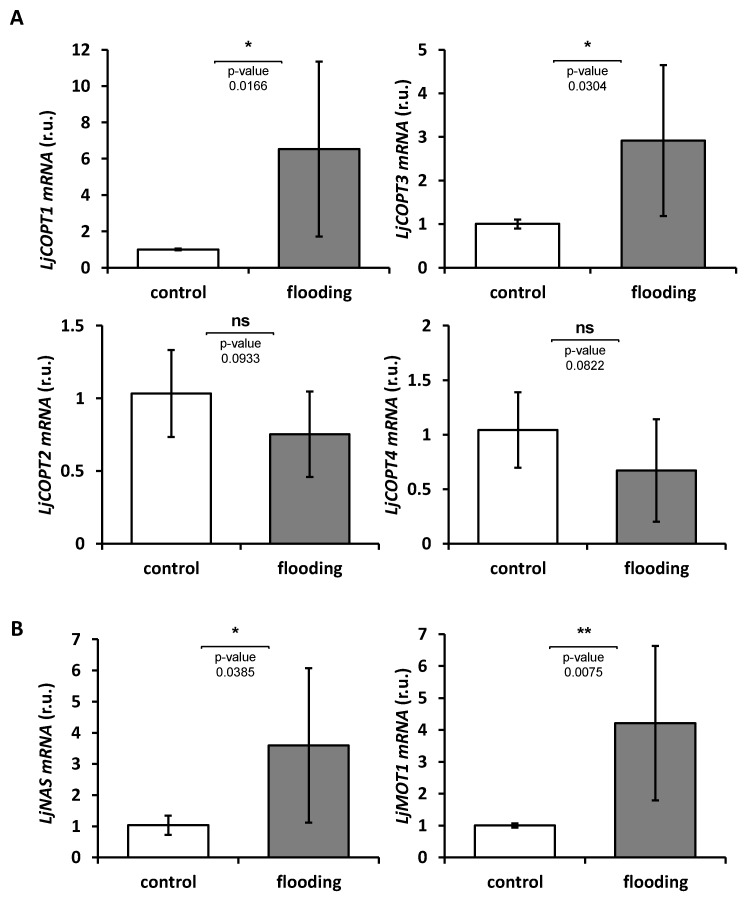
Gene expression of metal homeostasis related genes in *Lotus japonicus*. Gene expression of *LjCOPTs* (**A**), *Lotus japonicus Nicotianamine Synthase* (*LjNAS*) and *L. japonicus Molybdenum transporter LjMOT1* (**B**) were analyzed by RT-qPCR from shoots and normalized to the transcript levels of *EF*. The control condition was used as a reference. Plants were grown under partial submergence as described in Material and Methods. Values are means ± SD of three biological replicates. Between treatments for each genotypes, one asterisk (*P* < 0.05) and two asterisks (*P* < 0.01) over the bars mark a significant difference; ns indicate no significant difference (*P* > 0.05).

In order to evaluate whether gene expression was affected by the partial submergence treatment, the relative expression levels of *LjCOPT1–4* genes was determined in shoots samples by RT-qPCR. Partial submergence treatment differentially affected the relative expression of *LjCOPTs* (Figure 6A). *LjCOPT1* and *LjCOPT3* gene expression was up-regulated 6 and 3 times, respectively, compared to control conditions (Figure 6A). No differences caused by the treatment were detected for *LjCOPT2* and *LjCOPT4* gene expression (Figure 6A).

To ascertain whether Cu could be translocated to the aerial part through the xylem, the expression of genes involved in the internal mobilization of metals, such as the *Nicotianamine Synthase* (*NAS*) (reviewed in [41]) was analyzed. The partial submergence treatment affected the expression of the *LjNAS* gene, being up-regulated compared to the control (Figure 6B). Finally, the expression of a gene involved in Mo transport, which encodes the *Molybdenum Transporter 1* (*LjMOT1*), was evaluated. This gene has been annotated as a homolog of the *A. thaliana MOT1* gene [42], whose levels have been described to increase upon Cu-deficiency in *Brassica napus* [43]. Under flooding treatment *LjMOT1* was also up-regulated in the evaluated *L. japonicus* plants (Figure 6B). 

## 3. Discussion

Hypocuprosis is one of the most important nutritional deficiencies within livestock production [5]. A diet that would provide sufficient Cu to the animal is an interesting alternative to alleviate this nutritional problem while avoiding overtreatments in healthy cattle. Copper levels in forage depend on the species and the type of soil where they have grown. Legume species produce forage with better nutritional values than grasses and they accumulate higher Cu levels [44,45]. In addition, plant Cu uptake could be affected by certain soil conditions, such as redox potential, pH, and organic matter content [46].

In the last years, due to the extensive agronomical land uses, livestock are becoming relegated to marginal areas for agriculture. In these areas, it is more difficult for the implantation of forage legumes to occur, due to the presence of different abiotic stresses, like salinity, alkalinity, and flooding [47]. Also, areas designated for livestock are frequently subject to flooding, which due to climate change has increased considerably around the world in the last decades [48]. An excellent example of this fact occurs in cattle production systems in Flooding Pampa (Argentina), where only few legumes are present in natural pastures used as forage, with *Lotus* spp. playing a key role in the agrosystem’s sustainability [47,49].

At the Flooding Pampa area, flooding decreases the productivity of natural pastures by reducing plant growth and hence the forage biomass [6]. *Lotus* spp. is relatively tolerant to this abiotic stress. However, it has been described that its performance is reduced by hypoxia conditions [50] and these effects were observed under our conditions for all accessions evaluated (Figure S1; Table S3). The higher relative tolerance to hypoxia observed in some species from the *Lotus* genus have been supported by the formation of anatomical features, such as aerenchym and adventitious roots, in order to aid adequate oxygen diffusion [9,50]. In our conditions, the treated plants developed adventitious roots and the thickening of the main stem base, mainly in the Lt and LtxLc accessions (Figure 1A). These modifications are supposed to allow aerenchym formation [51]. 

Flooding stress also causes different physiological changes in plants, among them the closing of stomata, as well as a decrease in photosynthesis [52]. Both effects were observed in *Lotus* spp. plants subjected to partial submergence, decreasing the net photosynthesis (NP) and the stomatal conductance (*gs*) in all the accessions evaluated (Figure 1C and Table S3). If NP and *gs* were negatively affected by the treatment, the performance of PSII was not affected in Lt, LtxLc, and LcD plants (Figure 1B and Table S3), probably indicating that these accessions did not show symptoms of PS damage. However, the maximum quantum yield of photosystem II (*F*v/*F*m) was affected by partial submergence treatment in Lj and LcT plants (Figure 1B).

Taken together, our results indicate that Lt, LtxLc, and LcD plants display better performance under partial submergence treatment than Lj and LcT, as previously suggested [9,11]. The relatively worse performance of Lj and LcT could be associated with a lower endogenous Cu content in treated plants compared to their control (Figure 2A). Indeed, Cu levels in shoots from Lj and LcT treated plants were sufficiently low (around 1 µg/g DW) to be considered Cu-deficient, since the optimal Cu level for plants is above 5 µg/g DW [1]. Upon Cu limitation, the electron transport chain of chloroplasts is compromised as Cu is required for the synthesis of quinones of PSII (Baszynski et al. 1978), and it is essential for cuproproteins, such as plastocyanin, involved in the activity of PSI [53]. Thus, Cu deficiency could explain the low *F*v/*F*m values observed in Lj and LcT treated plants.

Transporters of the COPT family involved in the specific translocation of Cu are induced by the deficiency of this metal [22]. To evaluate Cu transporters in *Lotus* spp., *L. japonicus* was chosen for two main reasons: a) It is a model species whose genome has been completely sequenced [54]; and b) in response to partial submergence, its Cu levels are markedly reduced, leading to Cu deficiency (Figure 2A). Previously, the relevance of the COPT family of transporters has been evaluated in other species of agronomic interest, such as rice, maize, or vine [35,36,37,55]. In legumes, COPT members have been predicted by in silico analysis for *Glycine max*, *Phaseolus vulgaris*, and *Medicago truncatula*, among others. However, only MtCOPT1 from *M. truncatula* has been functionally characterized [13]. Here, we have identified and characterized four COPT members in *L. japonicus*, namely *LjCOPT1*, *LjCOPT2*, *LjCOPT3*, and *LjCOPT4*.

The predicted LjCOPT1 protein is phylogenetically close to the MtCOPT1 and MtCOPT2 proteins (Figure 3A). *MtCOPT1*, recently characterized in *M. truncatula*, is expressed specifically in nodules, and a knockout *copt1-1* mutant displayed a reduced nitrogen fixation rate [13]. These authors suggest that MtCOPT1 could be responsible for transporting Cu from the apoplast to the nodule and the same function could be postulated for LjCOPT1. In fact, transcriptome comparative analysis performed using the *Lotus japonicus* Gene Expression Atlas (LjGEA) database showed a two times greater expression of *LjCOPT1* in 21 day-old nodules than in uninoculated roots (Table S5). However, it is worth noting that *M. truncatula* forms an indeterminate-type of nodule, meanwhile *L. japonicus* form a determinate-type of nodule [56]. The role of COPT1 in both species could be different and more experiments would be required to establish its role in determinate-type nodule symbiosis.

The predicted LjCOPT3 and LjCOPT4 proteins were phylogenetically close to AtCOPT5 protein (Figure 4B). In *A. thaliana*, upon severe Cu deficiency, COPT5 facilitates Cu mobilization from root vacuoles to vegetative tissues in the shoots [30,57]. Moreover, AtCOPT5 participates in the interconnected mobilization of vacuolar Cu and Fe pools to fulfill interorgan metal translocation [34]. Finally, LjCOPT2 is the closest relative to the remaining COPT members of *A. thaliana* (Figure 3A). These members have been associated with the Cu influx into the cytosol; AtCOPT1-2-6 proteins act through the plasma membrane, meanwhile AtCOPT3 is located in intracellular compartments’ membranes [25,26,29,32,33].

*LjCOPT1* was highly expressed in the roots (Figure 5A) and similar results were obtained from the LjGEA database, where normalized transcript levels are 35-fold higher in roots than in shoots from 28 day-old plants (Table S2). At the same time, *LjCOPT2* and *LjCOPT3* were also expressed in the roots of *L. japonicus* plants and their relative expression was similar in stems (Figure 5A). In particular, normalized transcript levels for *LjCOPT3* obtained from the LjGEA database showed that this gene was highly expressed in all the tissues and principally in roots (Table S2). Accordingly, with the relative expression of these *COPT* genes, the Cu levels were higher in the roots (Figure 3B). Finally, the relative expression of *LjCOPT4* was higher in leaves (Figure 3A and Table S2).

Considering the relative expression levels, the role and the distribution of different LjCOPTs could be tissue-specific as follows: *LjCOPT1* in roots, *LjCOPT2* and *LjCOPT3* in roots and stems, and *LjCOPT4* in leaves (Figure 5A). The characterization of gene expression profiling by tissues allows a focus to be placed on specific LjCOPT members for biofortification strategies aimed to increase root Cu uptake capacity and translocation activity. Increasing the gene expression of copper transporters could provide additional micronutrients for vegetative tissue [58]. For example, transgenic plants overexpressing *COPT1* from *A. thaliana* increased endogenous Cu levels [29].

Under partial submergence, the expression of *LjCOPT1* and *LjCOPT3* was higher than under control conditions, suggesting that both genes were induced by hypoxia. In this sense, Kropat et al. [59] described that *Chlamydomonas* spp. subjected to low oxygen induce the response to Cu deficiency mediated by the copper response regulator 1 (CRR1) transcription factor (TF). In *A. thaliana*, the functional homolog to CRR1 is the SPL7 TF, regulating *AtCOPTs* gene expression by binding CuRE motifs [27]. CuREs motifs were detected in all the *LjCOPT*s promoters evaluated (Figure S2), which suggest a putative regulation under Cu deficiency and maybe hypoxia in *Lotus* spp.

Other specific responses to low oxygen have been described in higher plants compared to *Chlamydomonas* [60]. The analysis of the *LjCOPTs* promoter region (1000 bp upstream of the start codon) showed that anaerobic response elements (AREs motifs) [61] were present (Figure S2). However, the hypoxia-responsive promoter element (HRPE), which is necessary and sufficient for Ethylene Response Factor VII (ERF-VII) transactivation under low oxygen conditions [62], was not detected in *LjCOPTs* promoter sequences (Figure S2). Other motifs involved in the hypoxia response, such as two GT-motifs and a GC-box motif [63,64], were also detected in the *LjCOPT2* and in the *LjCOPT3* and *LjCOPT4* promoters, respectively (Figure S2). Recently, under hypoxia in humans, Cu availability has been shown to regulate target gene selectivity of the hypoxia-inducible factor (HIF-1α) transcription factor, affecting the binding to hypoxia response elements (HREs) in the promoters of some differential genes [65]. Taken together, these results suggest a complex scenario of transcriptional regulation under Cu deficiency and hypoxia conditions, where *cis*-regulatory elements present in the *Lotus* promoters participate differentially in *COPT* gene expression.

Members of the COPT family, such as the COPT2 transporter of *A. thaliana*, have been described to participate in Fe deficiency responses [25]. Despite the reduction in Cu levels caused by partial submergence in Lj and LcT, the levels of Fe were not significantly affected. However, Fe levels were positively affected by treatment in LtxLc and LcD plants (Figure 2B). Flooding conditions could increase Fe content in plants to even reach toxicity levels (up to 700 µg/g DW) [1], although, in our experimental conditions, the maximum values did not exceed 400 µg/g DW (Figure 2B). Once the Cu and Fe are incorporated by the root, both can be mobilized as metal–nicotianamine (NA) complexes from the roots to leaves [40,41,66]. In rice, overexpression of the *NAS3* gene increased Fe, Zn, and Cu content in seeds [67]. The fact that *LjNAS* expression increased under treatment (Figure 6B) suggests the mobilization of both metals to the sink tissues may be experiencing competition with NA. If this is the case, the restoration of appropriate Cu levels could also help to equilibrate Fe long distance transport in *Lotus* spp.

Flooding conditions also increase Mo levels in plants [68] and, in agreement, all *Lotus* accessions showed an enhancement in Mo levels caused by the treatment (Figure 2C). Despite Mo being highly required by legumes, Mo excess in a forage diet (up to 5 to 10 mg/Kg DW) can produce molybdenosis, further affecting Cu absorption, and being counterproductive for ruminant production [5]. Molybdenum is also associated with Cu homeostasis in plants since the Mo cofactor (MoCo) needs Cu for its biosynthesis [69]. Under flooding conditions, Lj*MOT1* gene expression was also induced (Figure 6B) accordingly with the high level of Mo concentration detected in all the *Lotus* spp. evaluated (Figure 1B). The increase of *MOT1* expression and Mo accumulation under Cu deficiency has also been reported in *Brassica napus* [43] and in legumes, and Mt*MOT1* is the only MOT family member described specifically in nodules and is responsible for Mo incorporation to the nodule cells [70].

From an agronomical point of view, and considering the levels of the three nutrients evaluated, the forage produced by Lt accession under partial submergence seems to be more adequate to an animal diet because Cu and Fe levels remained unaffected and Mo levels were relatively lower compared with the other accessions evaluated. However, Cu levels were under-optimal supply to fulfill cattle feeding requirements (Figure 2A). In this sense, the initial LjCOPT characterization in the present work could serve to design improved cultivars that accumulate optimal metal levels under marginal environments. 

## 4. Material and Methods

### 4.1. Plant Material and Treatments

For *COPTs* gene cloning and the relative expression assays, seeds from *L. japonicus* ecotype MG20 (from Faculty of Agriculture, University of Miyazaki, Miyazaki, Japan) were scarified (as reported in Escaray et al. [71]) and sown in 250 cc pots containing sand:perlite (1:3) substrate. Plants were cultivated in grown chambers under optimal conditions as described in Escaray et al. [71] and irrigated with Hoagland 0.5X [72]. One-month old plants were harvested and separated in tissues (leaves, stems, and roots) and samples were immediately frozen in liquid nitrogen and stored at –80 °C for DNA and RNA isolation.

For partial submergence assays, seeds from two accessions of *L. corniculatus* were used (a commercial tetraploid cv. “San Gabriel” (LcT) and a natural diploid accession “charlii” (LcD) as described in Escaray et al. [71]. Moreover, one accession from *L. tenuis* (Lt), collected from naturalized populations at Chacra Experimental Integrada Chascomús—INTA, Buenos Aires (Argentine), an interspecific hybrid *L. corniculatus* × *L. tenuis* (LtxLc) [71], and *L. japonicus* MG20 ecotype (Lj) were used in the assays. Seeds were scarified, sowed, and incubated in a growth chamber under similar light, temperature, and humidity conditions described by Antonelli et al. [9]. Then, seedlings were transferred to 4 L pots containing sand:soil (1:1) and cultivated in a greenhouse. Assay was performed between spring–summer of 2014–2015 (location: 35°37′47″ S, 57°59′50″ W, Chascomús, Province of Buenos Aires, Argentina). The mean temperature was 23 ± 5 °C, and natural irradiance per day was 1100 ± 250 μmol m^−2^·s^−1^. Irrigation was performed with water from a rainwater harvesting system. Partial submergence (flooding) treatment was applied on 30 day-old plants during 6 weeks. This condition was achieved by obstructing drainage and adding water until it was 6 cm above the substrate surface. For the control, plants were cultivated under similar conditions with periodical irrigation and free drainage, maintaining the humidity at not less than 80% of the field capacity.

### 4.2. Fluorescence and Gas Exchange Measurements

Photosystem II (PSII) activity and performance index (Pi) were estimated by non-invasive OJIP tests [73]. Measurements were performed at the end of the treatment on the third fully expanded leaf (Pocket PEA Chlorophyll Fluorimeter, Hansatech Instruments, UK) [9].

A Portable Photosynthesis System (PP System TPS 2, Amesbury, MA 01913, USA) was used to determinate the net photosynthetic rate (*Asat*) and stomatal conductance (*gs*). Measurements were taken one day before the end of partial submergence treatment on the fifth fully expanded leaf from apex using an LED light unit at saturating irradiance intensity (1500 μmol m^2^·s^−1^).

### 4.3. Copper, Iron, and Molybdenum Determination 

For metal content determination, 10 to 20 mg of fresh tissue from *Lotus* spp. shoots were dried at 70 °C until a stable weight and digested with 500 µL of HNO_3_ at 90 to 110 °C. Digested samples were diluted with 1.5 to 2 mL ultrapure H_2_O. The Cu and Fe content was analyzed using a Buck Scientific VGP 210 Atomic Absorption Spectrophotometer (E. Norwalk, CT, USA) by the electrothermal atomization method using pyrolytic graphite tubes at the Departamento de Química Analítica Instrumental at the Universidad de Buenos Aires. Magnesium nitrate (Mg(NO_3_)_2_, 20 µL 1000 ppm), was used as a matrix modifier for Fe determination. Molybdenum content was analyzed by inductively coupled plasma mass spectroscopy, ICP-MS (Agilent model 7900, Santa Clara, CA, USA), at the Servei Central de Suport a la Investigació Experimental (S.C.S.I.E) at the Universitat de València.

### 4.4. Plasmid Constructs 

The *COPTs* coding sequence was amplified from *L. japonicus* cDNA samples using specific primers detailed in Table S1 and subcloned into the BamHI/HindIII restriction enzyme site of the yeast multicopy expression vector p426GPD [74].

### 4.5. Functional Complementation Experiments in Yeast 

MPY17 (MATa, *ctr1::ura3::KanR*, *ctr3::TRP1*, *his3*, *lys2-802*, *CUIP1R*) cells transformed with p426GPD plasmid containing *AtCOPT1* or the four *LjCOPTs* were grown in synthetic complete medium without uracil (SC-ura) to OD_600_ = 1.0. MPY17 cells transformed with the empty vector were used as a negative control. Two 10-fold serial dilutions were plated on SC and SC-Ura, SC plus Ferrozine (300 µM), E/G (2% ethanol, 3% glycerol), or E/G plus Cu (100 µM CuSO_4_). Plates were incubated for 3 (SC media) or 10 days (−Fe, E/G, and E/G + Cu media) at 30 °C and photographed.

### 4.6. RNA Isolation and Gene Expression by Real-Time qPCR

Total RNA was extracted from different tissue samples with a Plant Spectrum Total RNA Kit (Sigma, Darmstadt, Germany) following the manufacturer’s instructions. RNA quality was checked on agarose gel electrophoresis and treated with DNase I Amp Grade (Invitrogen, Waltham, MA, USA). The null PCR amplification of the primer pair, ITS1/ITS4 [71], was used to confirm the absence of DNA contamination from the RNA samples. Obtained samples were quantified by UV spectrophotometry and 2 µg of RNA was used to perform the cDNA synthesis by the reverse transcription reaction using SuperScript III kit (Invitrogen, Waltham, MA, USA) according to the supplier’s instructions.

Specific primers were designed to evaluate *LjCOPT1-4* gene expression (Table S2); alpha *ELONGATION FACTOR* (*EF-1α*) gene was utilized as housekeeping [71]. The primer pairs were initially checked by RT-qPCR for their specificity by dissociation analysis. In turn, amplification efficiency was assessed by performing a standard curve for each gene using six dilution points.

An aliquot of 5 μL of 1:10 diluted cDNA and 2.5 pmol of each primer were used in the PCR reaction. The SYBR-Green qPCR Super-Mix-UDG with ROX (Invitrogen, Waltham, MA, USA) was used according to the supplier’s instructions in 20 μL of the final volume. Three biological replicates were performed per sample and gene. Cycling parameters were two initial steps of 50 °C for 2 min and 95 °C for 2 min, a two-step cycle of 95 °C for 15 s and 60 °C for 1 min repeated 50 times, and a final step of 10 min at 60 °C plus the dissociation curve. Amplifications were performed on a CFX96 Touch Real Time PCR Detection System (Bio-Rad, Hercules, CA, USA). For each transcript, the average threshold cycle (Ct) was determined. The gene quantification method based on the relative expression of the target gene versus the reference gene, *EF-1α*, was adopted.

### 4.7. Bioinformatics and Statistical Analysis

*L. japonicus* COPT family members were identified in the *Lotus japonicus* genome assembly build 2.5 site (http://www.kazusa.or.jp/lotus/release2/index.html). All sequences are full CDS, except for *chr1.LjT44L17.30.r2.d* (*LjCOPT1*), whose available sequence is partial. To obtain the full CDS of the *LjCOPT1* gene a, in silico analysis of *L. japonicus* Illumina reads (available at the NCBI BioProject browser, accession number PRJNA288510) was performed to obtain the specific primer pair to amplify its gene (Table S1). The obtained PCR product was sequenced and deposited in the Gene Bank under the following number: MN065182. Sequences from model COPT proteins were obtained from TAIR (https://www.arabidopsis.org/), JCVI (http://www.jcvi.org/medicago/index.php), Rice Genome Annotation Project (http://rice.plantbiology.msu.edu), and Uniprot (http://www.uniprot.org): *Medicago truncatula* (MtCOPT1 to MtCOPT8: Medtr4g019870, Medtr7g066070, Medtr3g105330, Medtr4g0 64963, Medtr4g065660, Medtr1g015000, Medtr4g065123, Medtr0027s0220), *Arabidopsis thaliana* (AtCOPT1 to AtCOPT6: At5g59030, At3g46900, At5g59040, At2g37925, At5g20650, At2g26975), *Oryza sativa* (OsCOPT1 to OsCOPT7: Os01g56420, Os01g56430, Os03g25470, Os04g33900, Os05g35050, Os08g35490, Os09g26900), *Brachypodium distachion* (BdCOPT1 to BdCOPT5: Bradi1g24180, Bradi1g24190, Bradi2g51210, Bradi4g31330, Bradi5g09580), *Glycine max* (GmCOPT1 to GmCOPT9: Glyma_11g134700, Glyma_18g191300, Glyma_04g057000, Glyma_06g057400, Glyma_01g106700, Glyma_07g141200, Glyma_07g141600, Glyma_14g107100, Glyma_17g219400, Glyma_18g191900), and *Phaseolus vulgaris* (PvCOPT1 to PvCOPT6: Phavu_011 g060400g, Phavu_011g060500g, Phavu_008g112800g, Phavu_009g083400g, Phavu_008g113200g, Phavu_009g083400g).

Multiple sequence alignment was conducted in MUSCLE (Multiple Sequence Comparison by Log-Expectation) with default parameters. The evolutionary history was inferred by using the maximum likelihood method and the Jones-Taylor-Thornton (JTT) matrix-based model [75]. The tree with the highest log likelihood (–9636.67) is shown. The initial tree for the heuristic search was obtained automatically by applying the neighbor-join and Bio neighbor-join algorithms to a matrix of pairwise distances estimated using a JTT model, and then selecting the topology with the superior log likelihood value. Analysis involved 46 amino acid sequences. There was a total of 274 positions in the final dataset. Evolutionary analyses were conducted in MEGA X [76].

The determination of distinct motifs in the promoters of genes was performed using the PLACE (Plant Cis-Acting Regulatory DNA Elements) database (http://www.dna.affrc.go.jp/PLACE/).

Statistical analysis of relative expression was performed by comparing the relative expression of the genes based on the pairwise fixed reallocation randomization test (*P*-value < 0.05; [77]). Statistical analysis for the other parameters was carried out using two-way ANOVA with the means compared by the Duncan test (*P*-value < 0.05) or Kruskal–Wallis test (*P*-value < 0.05) for non-parametric data using the InfoStat software (version: Infostat/L, Universidad Nacional de Córdoba, Córdoba, Argentina) [78].

## Supplementary Materials

Supplementary materials can be found at https://www.mdpi.com/1422-0067/20/13/3136/s1.
