# Supplementary material for: Characterization of the Copper Transporters from Lotus spp. and Their Involvement under Flooding Conditions
Source: Int J Mol Sci. 2019 Jun 27;20(13):3136. doi: 10.3390/ijms20133136 (PMC6651048; doi:10.3390/ijms20133136)
Supplement: Supplementary file 1 [file ijms-20-03136-s001.pdf]

Table S1. Sequence of the oligonucleotides used for plasmid constructs.

**LjCOPT1 Fw**	5'	CATGGATCCATGGACATGCCCATGTCTAAC	3'
**LjCOPT1 Rv**	5'	CATAAGCTTTCAAATTTTGAGAGTAGCAGAGGAGGAC	3'
**LjCOPT2 Fw**	5'	CATGGATCCATGGAACCATCTTTATCAACAAG	3'
**LjCOPT2 Rv**	5'	CATAAGCTTCTACAGTTTTGAAGAATCCAATCCG	3'
**LjCOPT3 Fw**	5'	CATGGATCCATGATGCACATGACCTTTTACTGG	3'
**LjCOPT3 Rv**	5'	CATAAGCTTTCAAGCACAAGCGCAGG	3'
**LjCOPT4 Fw**	5'	CATGGATCCATGATGAAAATGACATTGCATTGGAG	3'
**LjCOPT4 Rv**	5'	CATAAGCTTCTAAGCGCATGCACAAGAATC	3'

Table S2. Sequence of the oligonucleotides used for qPCR.

**qLjCOPT1 Fw**	5'	GGAAAACATGCCACAGTGCT	3'
**qLjCOPT1 Rv**	5'	CGATAAATCTCAGTCGTAATGGC	3'
**qLjCOPT2 Fw**	5'	TGGGGCTACAAGGTGAACAT	3'
**qLjCOPT2 Rv**	5'	ACCAAGTAAGAAAGCCCGGA	3'
**qLjCOPT3 Fw**	5'	GCTGGAGATTGAAACCCCTC	3'
**qLjCOPT3 Rv**	5'	CACAAACACCCCACCGTTAA	3'
**qLjCOPT4 Fw**	5'	GTTTTGTTCGGGGTGGACTC	3'
**qLjCOPT4 Rv**	5'	TCAACTTCAACTGCAGCACC	3'

Table S3. Effect of partial submergence on stomatal conductance (*gs*: mmol H_2O m^{-2} s^{-1}), performance index (PIabs) and biomass production. Lt: *L. tenuis*; LtxLc: interspecific hybrid *L. tenuis x L. corniculatus* diploid; LcD: *L. corniculatus* diploid; LcT: *L. corniculatus* tetraploid and Lj: *L. japonicus*. *P*-value correspond to control vs. flooding treatment comparison (T-test).

**Genotype**	**Stomatal conductance (*gs*)**						
	**Control (mean ± SD)**			**Flooding (mean ± SD)**			***P*-value**
**LcD**	457.89	±	72.88	400.30	±	186.30	0.4968
**LtxLc**	612.64	±	212.46	272.25	±	110.47	**0.0059**
**Lt**	611.05	±	292.10	253.23	±	124.79	**0.0202**
**Lj**	554.75	±	178.24	52.49	±	18.82	**0.0010**
**LcT**	499.66	±	287.23	27.56	±	20.49	**0.0102**

**Genotype**	**Performance index (PIabs)**						
	**Control (mean ± SD)**			**Flooding (mean ± SD)**			***P*-value**
**LcD**	1.81	±	1.39	0.81	±	1.05	0.0957
**LtxLc**	3.34	±	1.70	1.05	±	0.58	**0.0103**
**Lt**	2.97	±	1.82	1.83	±	0.84	0.0973
**Lj**	1.33	±	1.29	0.17	±	0.17	**0.0403**
**LcT**	2.85	±	1.86	0.26	±	0.34	**0.0102**

**Genotype**	**Biomass**						
	**Control (mean ± SD)**			**Flooding (mean ± SD)**			***P*-value**
**LcD**	3.38	±	0.70	0.95	±	0.31	**<0.0001**
**LtxLc**	6.16	±	1.78	2.12	±	0.47	**0.0009**
**Lt**	7.27	±	1.67	2.26	±	0.63	**<0.0001**
**Lj**	4.14	±	0.87	1.48	±	0.43	**0.003**
**LcT**	8.15	±	2.21	2.39	±	0.65	**0.0001**

Table S4. Matrix of identity between COPT proteins.

	MtCOPT1	MtCOPT2	MtCOPT3	MtCOPT4	MtCOPT5	MtCOPT6	MtCOPT7	MtCOPT8
MtCOPT1	ID							
MtCOPT2	0.527	ID						
MtCOPT3	0.214	0.198	ID					
MtCOPT4	0.347	0.373	0.193	ID				
MtCOPT5	0.352	0.412	0.238	0.690	ID			
MtCOPT6	0.260	0.236	0.591	0.232	0.258	ID		
MtCOPT7	0.282	0.331	0.192	0.581	0.587	0.218	ID	
MtCOPT8	0.324	0.346	0.210	0.351	0.382	0.213	0.326	ID
AtCOPT1	0.325	0.345	0.223	0.427	0.452	0.234	0.347	0.341
AtCOPT2	0.318	0.371	0.232	0.391	0.431	0.247	0.329	0.331
AtCOPT3	0.311	0.352	0.224	0.349	0.365	0.238	0.346	0.383
AtCOPT4	0.292	0.314	0.182	0.303	0.305	0.209	0.255	0.388
AtCOPT5	0.243	0.209	0.529	0.229	0.254	0.554	0.220	0.244
AtCOPT6	0.360	0.375	0.231	0.397	0.425	0.251	0.346	0.375
OsCOPT1	0.300	0.327	0.236	0.389	0.420	0.245	0.317	0.298
OsCOPT3	0.301	0.341	0.234	0.384	0.436	0.267	0.337	0.329
OsCOPT4	0.256	0.306	0.176	0.304	0.299	0.175	0.262	0.275
OsCOPT6	0.254	0.313	0.191	0.342	0.329	0.191	0.264	0.243
OsCOPT2	0.245	0.327	0.226	0.350	0.368	0.238	0.318	0.289
OsCOPT5.2	0.202	0.213	0.385	0.201	0.227	0.361	0.201	0.198
OsCOPT5.1	0.220	0.193	0.572	0.224	0.259	0.516	0.244	0.233
GmCOPT1	0.379	0.373	0.193	0.696	0.656	0.223	0.541	0.387
GmCOPT2	0.510	0.587	0.191	0.345	0.379	0.223	0.339	0.346
GmCOPT3.1	0.202	0.192	0.762	0.204	0.222	0.571	0.192	0.198
GmCOPT3.2	0.208	0.198	0.776	0.214	0.233	0.578	0.192	0.204
GmCOPT4	0.564	0.617	0.196	0.349	0.396	0.216	0.311	0.305
GmCOPT5	0.514	0.578	0.187	0.331	0.354	0.233	0.310	0.337
GmCOPT6	0.337	0.309	0.245	0.363	0.382	0.219	0.333	0.697
GmCOPT7	0.228	0.201	0.673	0.206	0.224	0.666	0.212	0.230
GmCOPT8	0.233	0.194	0.668	0.205	0.222	0.702	0.211	0.212
GmCOPT9	0.320	0.316	0.230	0.363	0.388	0.205	0.326	0.774
PvCOPT1	0.320	0.300	0.210	0.575	0.551	0.229	0.455	0.339
PvCOPT2	0.314	0.314	0.217	0.587	0.557	0.242	0.461	0.335
PvCOPT3	0.290	0.298	0.208	0.355	0.365	0.195	0.302	0.718
PvCOPT4	0.196	0.187	0.762	0.198	0.233	0.564	0.187	0.198
PvCOPT5	0.490	0.601	0.195	0.365	0.382	0.225	0.311	0.299
PvCOPT6	0.196	0.187	0.762	0.198	0.233	0.564	0.187	0.198
**LjCOPT1**	**0.557**	**0.696**	**0.214**	**0.337**	**0.367**	**0.242**	**0.311**	**0.333**
**LjCOPT2**	**0.255**	**0.240**	**0.176**	**0.317**	**0.314**	**0.202**	**0.259**	**0.574**
**LjCOPT3**	**0.251**	**0.245**	**0.762**	**0.230**	**0.250**	**0.659**	**0.222**	**0.238**
**LjCOPT4**	**0.215**	**0.198**	**0.549**	**0.188**	**0.222**	**0.613**	**0.198**	**0.211**
BdCOPT1	0.245	0.289	0.166	0.288	0.297	0.170	0.240	0.262
BdCOPT2	0.251	0.301	0.173	0.293	0.293	0.160	0.255	0.222
BdCOPT3	0.274	0.298	0.235	0.362	0.387	0.227	0.327	0.275
BdCOPT4	0.211	0.202	0.552	0.222	0.245	0.516	0.224	0.208
BdCOPT5	0.291	0.351	0.228	0.335	0.344	0.226	0.277	0.284

	AtCOPT1	AtCOPT2	AtCOPT3	AtCOPT4	AtCOPT5	AtCOPT6	OsCOPT1	OsCOPT3	OsCOPT4
AtCOPT1	ID								
AtCOPT2	0.662	ID							
AtCOPT3	0.441	0.462	ID						
AtCOPT4	0.358	0.358	0.346	ID					
AtCOPT5	0.212	0.251	0.245	0.211	ID				
AtCOPT6	0.576	0.708	0.473	0.401	0.235	ID			
OsCOPT1	0.405	0.389	0.345	0.347	0.241	0.409	ID		
OsCOPT3	0.403	0.372	0.341	0.339	0.268	0.383	0.571	ID	
OsCOPT4	0.316	0.288	0.247	0.237	0.175	0.282	0.317	0.374	ID
OsCOPT6	0.350	0.318	0.306	0.286	0.195	0.308	0.365	0.378	0.412
OsCOPT2	0.381	0.354	0.327	0.320	0.242	0.354	0.548	0.573	0.327
OsCOPT5.2	0.200	0.204	0.234	0.196	0.359	0.220	0.245	0.270	0.212
OsCOPT5.1	0.229	0.236	0.245	0.201	0.529	0.247	0.263	0.284	0.212
GmCOPT1	0.404	0.386	0.371	0.327	0.225	0.430	0.430	0.393	0.284
GmCOPT2	0.345	0.333	0.318	0.308	0.213	0.355	0.312	0.303	0.308
GmCOPT3.1	0.187	0.198	0.224	0.194	0.476	0.202	0.230	0.217	0.171
GmCOPT3.2	0.192	0.209	0.219	0.194	0.490	0.213	0.230	0.228	0.176
GmCOPT4	0.323	0.339	0.337	0.286	0.201	0.353	0.298	0.301	0.304
GmCOPT5	0.339	0.320	0.335	0.315	0.210	0.337	0.288	0.286	0.273
GmCOPT6	0.341	0.345	0.384	0.370	0.233	0.390	0.304	0.335	0.262
GmCOPT7	0.202	0.213	0.237	0.214	0.565	0.210	0.244	0.254	0.195
GmCOPT8	0.190	0.201	0.236	0.191	0.568	0.203	0.248	0.252	0.184
GmCOPT9	0.347	0.347	0.393	0.390	0.219	0.390	0.306	0.331	0.270
PvCOPT1	0.348	0.375	0.384	0.278	0.237	0.424	0.385	0.367	0.243
PvCOPT2	0.348	0.385	0.388	0.265	0.244	0.423	0.374	0.367	0.238
PvCOPT3	0.322	0.327	0.378	0.375	0.208	0.368	0.297	0.317	0.262
PvCOPT4	0.182	0.193	0.219	0.200	0.490	0.196	0.230	0.217	0.171
PvCOPT5	0.325	0.351	0.335	0.310	0.211	0.383	0.303	0.306	0.293
PvCOPT6	0.182	0.193	0.219	0.200	0.490	0.196	0.230	0.217	0.171
**LjCOPT1**	**0.339**	**0.352**	**0.352**	**0.314**	**0.225**	**0.362**	**0.327**	**0.341**	**0.300**
**LjCOPT2**	**0.284**	**0.280**	**0.302**	**0.320**	**0.186**	**0.303**	**0.250**	**0.291**	**0.225**
**LjCOPT3**	**0.239**	**0.243**	**0.252**	**0.222**	**0.536**	**0.254**	**0.269**	**0.262**	**0.186**
**LjCOPT4**	**0.183**	**0.190**	**0.231**	**0.165**	**0.458**	**0.166**	**0.224**	**0.236**	**0.192**
BdCOPT1	0.322	0.297	0.295	0.267	0.165	0.322	0.291	0.340	0.473
BdCOPT2	0.317	0.288	0.276	0.243	0.166	0.298	0.294	0.317	0.456
BdCOPT3	0.359	0.351	0.321	0.279	0.227	0.369	0.541	0.586	0.364
BdCOPT4	0.222	0.234	0.227	0.209	0.542	0.244	0.265	0.281	0.200
BdCOPT5	0.362	0.325	0.348	0.279	0.205	0.345	0.348	0.388	0.455

	OsCOPT6	OsCOPT2	OsCOPT5.2	OsCOPT5.1	GmCOPT1	GmCOPT2	GmCOPT3.1	GmCOPT3.2	GmCOPT4	GmCOPT5
OsCOPT6	ID									
OsCOPT2	0.394	ID								
OsCOPT5.2	0.193	0.226	ID							
OsCOPT5.1	0.218	0.255	0.441	ID						
GmCOPT1	0.356	0.365	0.205	0.231	ID					
GmCOPT2	0.299	0.277	0.196	0.197	0.378	ID				
GmCOPT3.1	0.202	0.209	0.391	0.526	0.209	0.191	ID			
GmCOPT3.2	0.196	0.220	0.391	0.526	0.220	0.197	0.951	ID		
GmCOPT4	0.295	0.296	0.196	0.196	0.351	0.574	0.179	0.184	ID	
GmCOPT5	0.281	0.260	0.189	0.182	0.354	0.750	0.187	0.193	0.547	ID
GmCOPT6	0.248	0.283	0.213	0.257	0.393	0.346	0.233	0.239	0.324	0.364
GmCOPT7	0.196	0.236	0.405	0.562	0.217	0.205	0.687	0.693	0.204	0.196
GmCOPT8	0.190	0.235	0.388	0.555	0.216	0.198	0.682	0.682	0.209	0.201
GmCOPT9	0.264	0.285	0.201	0.247	0.406	0.341	0.219	0.224	0.320	0.337
PvCOPT1	0.297	0.339	0.205	0.254	0.735	0.320	0.193	0.204	0.292	0.283
PvCOPT2	0.302	0.351	0.212	0.266	0.741	0.320	0.206	0.212	0.312	0.301
PvCOPT3	0.259	0.272	0.193	0.230	0.396	0.311	0.208	0.213	0.309	0.312
PvCOPT4	0.207	0.220	0.385	0.500	0.204	0.191	0.895	0.902	0.179	0.187
PvCOPT5	0.313	0.286	0.210	0.206	0.368	0.703	0.195	0.201	0.567	0.640
PvCOPT6	0.207	0.220	0.385	0.500	0.204	0.191	0.895	0.902	0.179	0.187
**LjCOPT1**	**0.318**	**0.301**	**0.227**	**0.220**	**0.360**	**0.575**	**0.202**	**0.208**	**0.578**	**0.611**
**LjCOPT2**	**0.234**	**0.253**	**0.171**	**0.200**	**0.319**	**0.252**	**0.186**	**0.190**	**0.250**	**0.262**
**LjCOPT3**	**0.202**	**0.261**	**0.374**	**0.559**	**0.237**	**0.220**	**0.720**	**0.720**	**0.219**	**0.224**
**LjCOPT4**	**0.219**	**0.233**	**0.335**	**0.467**	**0.204**	**0.179**	**0.523**	**0.543**	**0.197**	**0.193**
BdCOPT1	0.405	0.304	0.201	0.193	0.319	0.300	0.151	0.156	0.273	0.273
BdCOPT2	0.405	0.297	0.185	0.169	0.310	0.301	0.163	0.168	0.296	0.256
BdCOPT3	0.408	0.470	0.247	0.270	0.406	0.295	0.213	0.219	0.289	0.274
BdCOPT4	0.226	0.264	0.491	0.821	0.223	0.184	0.513	0.506	0.194	0.174
BdCOPT5	0.521	0.374	0.238	0.244	0.355	0.333	0.222	0.222	0.339	0.305

	GmCOPT6	GmCOPT7	GmCOPT8	GmCOPT9	PvCOPT1	PvCOPT2	PvCOPT3	PvCOPT4	PvCOPT5	PvCOPT6
GmCOPT6	ID									
GmCOPT7	0.254	ID								
GmCOPT8	0.235	0.905	ID							
GmCOPT9	0.887	0.238	0.220	ID						
PvCOPT1	0.364	0.206	0.227	0.379	ID					
PvCOPT2	0.364	0.208	0.224	0.379	0.875	ID				
PvCOPT3	0.800	0.227	0.210	0.880	0.355	0.361	ID			
PvCOPT4	0.209	0.666	0.675	0.196	0.204	0.212	0.187	ID		
PvCOPT5	0.299	0.203	0.196	0.312	0.335	0.341	0.295	0.184	ID	
PvCOPT6	0.209	0.666	0.675	0.196	0.204	0.212	0.187	1.000	0.184	ID
**LjCOPT1**	**0.347**	**0.222**	**0.215**	**0.329**	**0.300**	**0.301**	**0.310**	**0.196**	**0.601**	**0.196**
**LjCOPT2**	**0.620**	**0.202**	**0.197**	**0.641**	**0.298**	**0.298**	**0.638**	**0.172**	**0.243**	**0.172**
**LjCOPT3**	**0.287**	**0.687**	**0.682**	**0.269**	**0.227**	**0.234**	**0.251**	**0.706**	**0.229**	**0.706**
**LjCOPT4**	**0.204**	**0.578**	**0.620**	**0.203**	**0.222**	**0.224**	**0.198**	**0.543**	**0.178**	**0.543**
BdCOPT1	0.267	0.180	0.174	0.289	0.278	0.267	0.265	0.151	0.284	0.151
BdCOPT2	0.251	0.176	0.180	0.255	0.270	0.259	0.233	0.168	0.266	0.168
BdCOPT3	0.303	0.242	0.235	0.311	0.365	0.365	0.305	0.213	0.298	0.213
BdCOPT4	0.231	0.542	0.555	0.222	0.234	0.247	0.207	0.467	0.209	0.467
BdCOPT5	0.255	0.236	0.229	0.280	0.319	0.320	0.272	0.217	0.343	0.217

	**LjCOPT1**	**LjCOPT2**	**LjCOPT3**	**LjCOPT4**	BdCOPT1	BdCOPT2	BdCOPT3	BdCOPT4	BdCOPT5
**LjCOPT1**	**ID**								
**LjCOPT2**	**0.256**	**ID**							
**LjCOPT3**	**0.245**	**0.209**	**ID**						
**LjCOPT4**	**0.202**	**0.181**	**0.549**	**ID**					
BdCOPT1	**0.278**	**0.235**	**0.161**	**0.191**	ID				
BdCOPT2	**0.290**	**0.209**	**0.163**	**0.180**	0.623	ID			
BdCOPT3	**0.304**	**0.263**	**0.245**	**0.208**	0.363	0.335	ID		
BdCOPT4	**0.217**	**0.180**	**0.552**	**0.442**	0.181	0.177	0.257	ID	
BdCOPT5	**0.363**	**0.236**	**0.244**	**0.230**	0.417	0.377	0.396	0.252	ID

Table S5. Transcriptome comparative analysis from *Lotus japonicus* Gene Expression Atlas (LjGEA) database.

Tissue	Treat. type	*LjCOPT1*	*LjCOPT2*	*LjCOPT3*	*LjCOPT4*	Ecotype	Reference
**Root (28 days-old plant)**	Standard	1042.7	107.0	8486.3	30.1	MG20	Verdier et al. 2013
**Stem (28 days-old plant)**	Standard	29.6	158.1	4461.2	29.5	MG20	Verdier et al. 2013
**Petiole (28 days-old plant)**	Standard	23.9	150.1	3437.0	39.7	MG20	Verdier et al. 2013
**Leaf (28 days-old plant)**	Standard	31.6	28.5	3290.6	117.8	MG20	Verdier et al. 2013
**Root 0h (uninoculated root)**	Low N	1297.1	75.2	7638.6	26.4	MG20	Verdier et al. 2013
**Nodule (Nod21 - 21 days post inoculation)**	Low N	2483.3	27.4	8833.0	35.8	MG20	Verdier et al. 2013
**WT Root Suseptible zone (3 week-old uninoculated plant)**	Standard	800.1	71.6	3650.8	38.8	Gifu	Høgslund et al. 2009
**WT Root tip (3 week-old uninoculated plant)**	Standard	111.5	58.7	1997.3	33.9	Gifu	Høgslund et al. 2009
**WT Root (3 week-old uninoculated plant)**	Standard	622.1	81.2	3312.9	30.3	Gifu	Høgslund et al. 2009
**WT Shoot (3 week-old uninoculated plant)**	Standard	40.3	37.0	2647.9	58.1	Gifu	Høgslund et al. 2009
**Pod10d (Pood+Seed)**	Standard	27.6	59.3	3941.0	69.2	MG20	Verdier et al. 2013
**Pod14d (Pood+Seed)**	Standard	85.5	24.3	5178.6	71.7	MG20	Verdier et al. 2013
**Pod20d (Pood+Seed)**	Standard	509.1	24.7	4057.7	49.8	MG20	Verdier et al. 2013
**Seed10d**	Standard	40.8	29.5	3551.3	29.8	MG20	Verdier et al. 2013
**Seed12d**	Standard	42.9	28.0	3073.9	28.5	MG20	Verdier et al. 2013
**Seed14d**	Standard	43.5	28.2	4321.4	31.4	MG20	Verdier et al. 2013
**Seed16d**	Standard	77.0	24.5	3974.7	35.6	MG20	Verdier et al. 2013
**Seed20d**	Standard	406.9	30.9	3932.2	38.7	MG20	Verdier et al. 2013

LjCOPT1 **(probeset: chr1.TM1284.2.1_at)**

ATGGACATGCCCATGTCTAACAGCACGATGCCGATGGAGATGCAGATGAGTTTCTATTGGGGAAAACATGCCACAGTGCTTTTTTCTGGGTGGCCTAACA
ACAGTGTTGGCATGTACATCTTAGCTCTCTTTTTTGTGTTCTCTTTAGCCATTACGACTGAGATTTATCGAAT

LjCOPT2 **(probeset: chr1.TM1284.2_at)**

ATGGAACCATCTTTATCAACAAGAATAATATCTTCATGTTCTATTCTTTCACGTCCCAACCTAACAGACAAGGTTTCCATGCATATATATAATGTGCAAA
GTACAAACCATTATCAATTTATACTACTCCATCACCTCAATATGTCGTACAACTCCACGGTGGCGACACGGCTGGGGCGACGGAGAATCCCCATACACAC
GACCCTCTACTGGGGCTACAAGGTGAACATACTGTTCCATTGCTGGCCCGGAAATAGCGCCGCCATGTACGCGGTGGCGCTAATGCTCGTGTTCGCCATG
GCGGTATTGGTGGAGTGGCTTTCCTTCACCAACATTGTGAAGCTCAAGCCCGGGGGTTCAAACCACGTCGTTGAGGCGCTCCTTAAGACGGGGCTCTACG
GCGTGCGTTCCGGGCTTTCTTACTTGGTAATGCTGGCCGTCATGTCGTTTAATGGCGGCGTTTTCCTCGCCGCAATTGGCGGCCACGTCATTGGTTTCTT
GATTTTCGGTACGCAGGCCTTCCGGAAGAAGTCATTAGGCGGATTGGATTCTTCAAAACTGTAG

LjCOPT3 **(probeset: chr1.CM0133.59_at)**

ATGATGCACATGACCTTTTACTGGGGGAAGAAGGTAACAATCCTCTTCGATTCATGGAAAACCGATTCATGGACGAGTTACATCCTGAGTCTACTCGCGT
GCTTCATCATCGCTGTTTTCTACCAGGTTCTCGAGAATCGCCGCATTCGCCTCAAGCTCTTCGCCGCCGGGAAGCCGGTTCCGCTGGAGATTGAAACCCC
TCTCATTCGCCGGAAGATTGTCGGAGATAAGGCGAAGGCTGGTGTGAAGGTTGGTGGATCGATTCTGTTCGGGGTGAGCTCGGGGATCGGGTATCTGCTC
ATGCTGGCGGTTATGTCGTTTAACGGTGGGGTGTTTGTGGCTATTGTGGTGGGGCTTGCGTTTGGGTATTTCTTCTTCAGGAGTGATGGGGAAGATTCTG
TTGTGGTTGATAGTTCCTGCGCTTGTGCTTGA

LjCOPT4 **(probeset: chr5.TM0455.6_at)**

ATGATGAAAATGACATTGCATTGGAGCAAAAGGGTCACCCTCCTCATAGATTCATGGGAGACCGATTCATGGCTGAGTTACATTCTCAGTTTACTCGCGT
GCCTCATGGTTTCCGCTTTCTACCAGTATCTAGAGAGTCTCCGGTTTCGGCTGAAGAGAGGGGAGTCTCCGGGGGCGGAGATACGGACGCCACTTCTGCG
GCGGAACAATGTTGCCGGGGGTAAGGTTGCTGAAGCGGTTTTGTTCGGGGTGGACTCTGCTGTTGGGTATTTGTTGATGTTGGCTATAATGTCATTCAAT
GGAGGGGTGTTTTTGGCTATTGTGGTGGGTCTTATGCTAGGTTACTTCTTCTTCAGGTGTCAGGGTGGAGACGGTGCTGCAGTTGAAGTTGACATTGATG
ATTCTTGTGCATGCGCTTAG

Here shows the full consensus sequence used during chip design (100 bp per line), in which the 11 25-mer probes are shown in **red** and **blue** alternatively. In case two probe sequences overlap, the overlapped portion is underlined. Also, the target sequence is shown in **bold** text. The target sequence normally starts from the first base of the first probe, and ends at the last base of the last probe

References

J. Verdier, I. Torres-Jerez, M. Wang, A. Andriankaja, S.N. Allen, J. He, et al., Establishment of the Lotus japonicus Gene Expression Atlas (LjGEA) and its use to explore legume seed maturation, Plant J. 74 (2013) 351–362.

N. Høgslund, S. Radutoiu, L. Krusell, V. Voroshilova, M.A. Hannah, N. Goffard, et al., Dissection of symbiosis and organ development by integrated transcriptome analysis of lotus japonicus mutant and wild-type plants, PLoS One. 4 (2009) e6556.

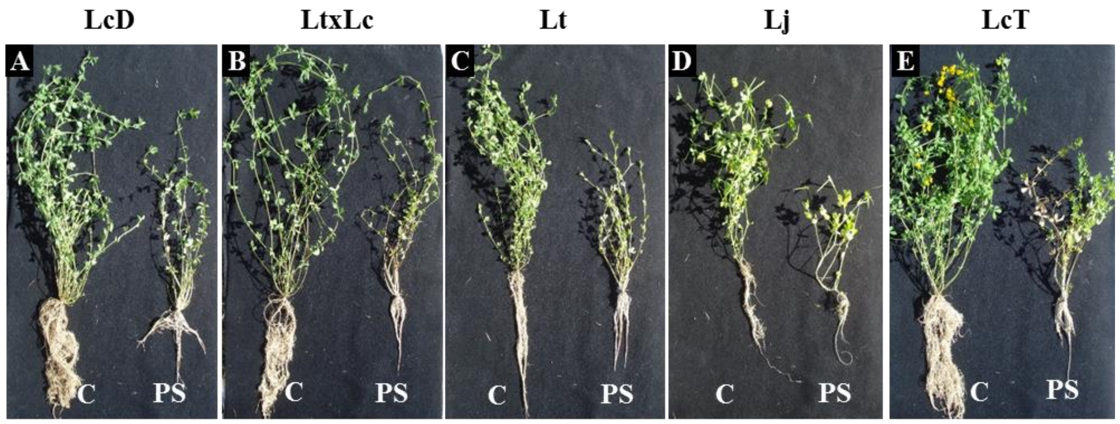

Figure S1. Effect of partial submergence in *Lotus* genus. Lt: *L. tenuis*; LtxLc: interspecific hybrid *L. tenuis x L. corniculatus* diploid; LcD: *L. corniculatus* diploid; LcT: *L. corniculatus* tetraploid and Lj: *L. japonicus*. Plants were grown as described in Figure 1; C: control. PS: partial submergence.

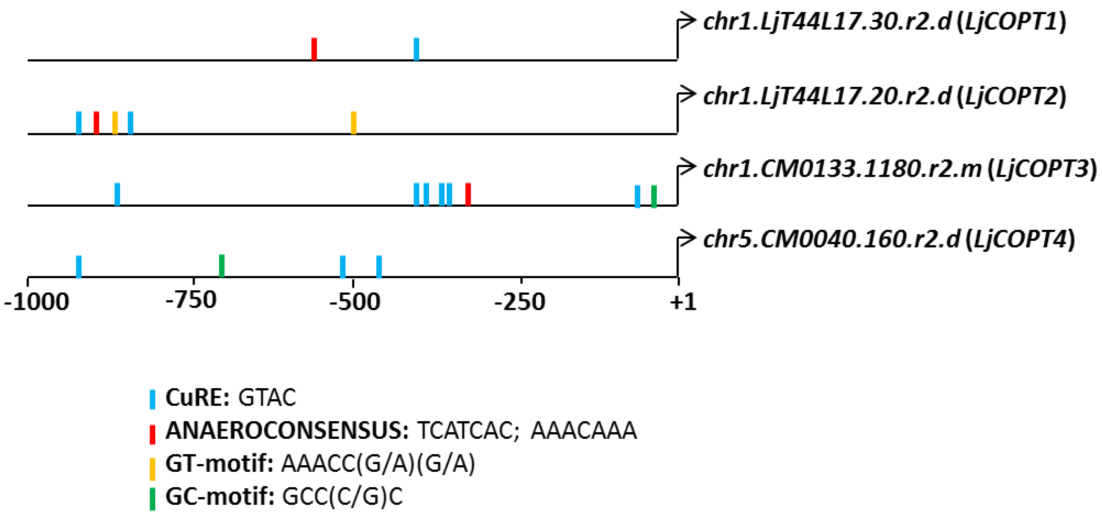

Figure S2. Sequence analysis of *LjCOPT1, LjCOPT2, LjCOPT3* and *LjCOPT4* promoters. Positions of putative *cis* regulatory elements are marked in different colors. Palindromic sequences are indicated only once. (References: Mohanty *et al*. 2005; Quinn and Merchant 1995; Hoeren 1998; Olive 1991).
